# TSG101, a tumor susceptibility gene, bidirectionally modulates cell invasion through regulating MMP-9 mRNA expression

**DOI:** 10.1186/s12885-015-1942-1

**Published:** 2015-11-25

**Authors:** Xu Bin Sai, Tomohiko Makiyama, Hiroshi Sakane, Yukimi Horii, Hideyuki Hiraishi, Hiromichi Shirataki

**Affiliations:** Department of Molecular and Cell Biology, Graduate School of Medicine, Dokkyo Medical University, 880 Kitakobayashi, Mibu-cho, Tochigi 321-0293 Japan; Department of Gastroenterology, Graduate School of Medicine, Dokkyo Medical University, 880 Kitakobayashi, Mibu-cho, Tochigi 321-0293 Japan; Present Address: Laboratory of Immunobiology, Faculty of Pharmaceutical Sciences, Fukuyama University, Sanzo Ichibanchi, Gakuencho, Fukuyama, Hiroshima 729-0292 Japan

**Keywords:** TSG101, Matrix metalloproteinases, Cell invasion, Tumor suppressor gene, Oncogene

## Abstract

**Background:**

Tumor susceptibility gene 101 (TSG101) was initially identified in fibroblasts as a tumor suppressor gene but subsequent studies show that TSG101 also functions as a tumor-enhancing gene in some epithelial tumor cells. Although previous studies have unraveled diverse biological functions of TSG101, the precise mechanism by which TSG101 is involved in carcinogenesis and tumor progression in a bidirectional and multifaceted manner remains unclear.

**Methods:**

To reveal the mechanism underlying bidirectional modulation of cell invasion by TSG101, we used RNA interference to examine whether TSG101 depletion bidirectionally modulated matrix metalloproteinase (MMP)-9 expression in different cell types.

**Results:**

TSG101 depletion promoted cell invasion of HT1080 cells but contrarily reduced cell invasion of HeLaS3 cells. In HT1080 cells, TSG101 depletion increased both baseline and phorbol 12-myristate 13-acetate (PMA)-induced MMP-9 secretion through enhancing MMP-9 mRNA expression, but did not affect the expression or activation of MMP-2. In contrast, TSG101 depletion decreased PMA-induced MMP-9 secretion through reducing MMP-9 mRNA expression in HeLaS3 cells. TSG101 depletion had little impact on the signaling pathways required for the activation of transcription of MMP-9 or MMP-9 mRNA stability in either cell line.

**Conclusion:**

TSG101 bidirectionally modulates cell invasion through regulating MMP-9 mRNA expression in different cell types. Our results provide a mechanistic context for the role of TSG101 in cell invasion as a multifaceted gene.

**Electronic supplementary material:**

The online version of this article (doi:10.1186/s12885-015-1942-1) contains supplementary material, which is available to authorized users.

## Background

Tumor susceptibility gene 101 (TSG101) was originally defined as a tumor suppressor gene and its functional inactivation in mouse fibroblasts produces cells with the capacity for colony formation in 0.5 % agar and the ability to form metastatic tumors in athymic nude mice [1]. TSG101 protein contains several unique domains, such as an inactive ubiquitin-conjugating domain, a proline-rich region, a coiled-coil domain, and a steadiness box [1, 2], implying diverse biological functions for TSG101 in endosomal trafficking [3–5], transcriptional regulation [6–10], and cell proliferation [11]. Recently, TSG101 has received attention in the field of exosome research because of its involvement in multivesicular body formation as a component of the endosomal sorting complex required for transport protein machinery [12–14]. Since inactivation of TSG101 leads to a series of mitosis-related abnormalities, TSG101 may be involved in genome stability [15, 16]. Moreover, since inactivation of TSG101 inhibits the endosomal trafficking of activated EGF receptors to the lysosome and thereby results in the prolonged induction of downstream signaling cascades, TSG101 may be involved in the negative regulation of receptor tyrosine kinase signaling [17, 18]. Thus, a series of studies are shedding light on the molecular mechanism by which TSG101 functions as a tumor suppressor gene. However, several recent reports have also demonstrated that TSG101 contrarily functions as a tumor-enhancing gene in some epithelial tumor cells [19, 20], suggesting that TSG101 may play divergent roles in carcinogenesis and tumor progression in different cell types.

Matrix metalloproteinases (MMPs) are a family of zinc-dependent proteolytic enzymes that degrade the extracellular matrix (ECM) [21]. This gene family consists of 23 members in human and is subdivided into two types: soluble MMPs and transmembrane-type MMPs. Most soluble MMPs are secreted from the cells as inactive zymogens that are activated on the cell membrane surface. Transmembrane-type MMPs are anchored to the cell membrane where they degrade the ECM and activate other MMPs. MMP activity is tightly regulated at the levels of transcription, activation of proenzymes by post-translational processes, and inhibition by endogenous proteins, i.e., tissue inhibitors of metalloproteinases (TIMP) [22]. However, in tumor invasion, excessive activity of MMPs breaks down the surrounding ECM microstructure, especially the basement membrane barrier, and thereby contributes to the invasion and migration of tumor cells [23–26]. Several studies have shown that among the many MMPs, gelatinases, especially MMP-2 and MMP-9, play a key role in degradation of type IV collagen, a major structural protein for the basement membrane barrier [27, 28]. Furthermore, an increase in expression of MMP-2 and MMP-9 has been reported in many human tumors including neuroblastoma and melanoma [29, 30]. MMP-2 is constitutively expressed in diverse cell types [31, 32]. In contrast, in most human tumors, MMP-9 expression is basally low but upregulated in response to diverse growth factors and cytokines [31–34]. Accumulating evidence suggests that the upregulation of MMP-9 expression contributes to the development of tumor progression such as invasion, metastasis, and angiogenesis [23, 26]. MMP-9 expression is regulated at several levels including gene transcription, mRNA stability, and translation [35–37]. A number of signaling pathways including the PKC, ERK, p38 kinase, JNK, PI3-K/Akt, and NF-κB signaling pathways are involved in the regulation of MMP-9 expression by various stimuli [38–45].

In this study, we observed that TSG101 depletion bidirectionally modulated cell invasion but not cell migration in different cell types. Among various molecules involved in cell invasion, we suspected that MMPs would be potential targets of TSG101 and used RNA interference (RNAi) to examine whether TSG101 depletion affects the expression of MMP-2 and MMP-9. We found that the regulation of cell invasion by TSG101 through modulating MMP-9 mRNA expression was bidirectional and multifaceted, and our findings unraveled a novel aspect of the functions of TSG101 in cell invasion as a multifaceted gene.

## Methods

### Cells, antibodies, and reagents

HT1080 and U2OS cells were purchased from the ATCC. HeLaS3 cells were a kind gift from Dr. Kishida (Kagoshima University Graduate School of Medical and Dental Sciences). HT1080 cells were grown in a humidified atmosphere of 5 % CO_2_ and 95 % air in Eagle’s minimal essential medium supplemented with 10 % fetal calf serum (FCS) (Invitrogen Corp., Carlsbad, CA, USA), 100 units/ml penicillin, and 100 mg/ml streptomycin at 37 °C. HeLaS3 and U2OS cells were grown in a humidified atmosphere of 5 % CO_2_ and 95 % air in Dulbecco’s modified Eagle’s medium supplemented with 10 % FCS (Invitrogen Corp.), 100 units/ml penicillin, and 100 mg/ml streptomycin at 37 °C. An anti-glyceraldehyde-3-phosphate dehydrogenase (GAPDH) antibody was purchased from MBL (Nagoya, Japan). Anti-MMP-9 and anti-TSG101 antibodies were purchased from Abcam (Cambridge, MA). Anti-Akt, anti-phospho-Akt (Ser473), anti-p38, anti-phospho-p38 (Thr180/Tyr182), anti-JNK, anti-phospho-JNK (Thr183/Tyr185), anti-NF-κB p65, anti-phospho-NF-κB p65 (Ser536), and anti-I-κB-α antibodies were purchased from Cell Signaling Technology (Danvers, CA). Anti-ERK and anti-phospho-ERK (Thr202/Ytr204) antibodies were purchased from BD Biosciences (San Jose, CA). PD98059, SB203580, SP600125, and LY294002 were purchased from Cell Signaling Technology. MG132 and bafilomycin A1 were purchased from Calbiochem (San Diego, CA) and Wako Pure Chemicals (Osaka, Japan), respectively.

### Transfection

Cells were transfected with 10 nM small interfering RNA (siRNA) targeting TSG101 or MMP-9 using RNAi max (Invitrogen Corp.) according to the manufacturer’s protocol. Negative control, TSG#1 (GCATGTACGTCTTCTGTCCCGTAAA), TSG#2 (GATACCCTCCCAATCCCAGTGGTTA), and MMP-9 (GGAAACCCTGCCAGTTTCCATTCAT) stealth siRNAs were purchased from Invitrogen.

### Western blotting

Cells were washed with cold phosphate buffered saline (PBS) and lysed with a lysis buffer [50 mM Tris/HCl pH 7.4, 150 mM NaCl, 1 × protease inhibitor cocktails (Roche Applied Science, Basal, Switzerland), 1 × phosphatase inhibitor mixture (Calbiochem), and 1 % NP-40] on ice for 10 min. Cell lysates were clarified by centrifugation at 10,000 × g for 10 min at 4 °C, and the supernatants were collected for western blotting. Protein lysate concentration was determined using the DC protein assay kit (Bio-Rad, Hercules, CA). The supernatants were separated by SDS-PAGE. Western blotting was performed using the ECL-Plus immunoblotting detection system (GE Healthcare UK Ltd., Amersham Place, England) in accordance with the manufacturer’s instructions. The relative density of each immunoreactive band was quantified using LAS-1000 plus gel documentation system (Fuji Film Co., Fuji, Japan).

### Wound-healing assay

Confluent cells were serum starved for 16 h prior to being carefully scratched using a 10 μl pipette tip. The cellular debris was subsequently removed by washing with PBS, and the cells were incubated in fresh serum-free medium containing or not 200 nM phorbol 12-myristate 13-acetate (PMA) (Sigma-Aldrich, St. Louis, MO). The cultures were photographed at 0, 3, or 9 h to monitor the migration of cells into the wounded area, and the width of the wounds is expressed as a percentage of the initial width at zero time.

### Invasion assay

At 48 and 24 h post-transfection, HT1080 and HeLaS3 cells, respectively, were trypsinized to detach cells from the culture dish, resuspended in medium containing 10 % FCS, washed twice with serum-free medium, and resuspended in serum-free medium containing 0.1 % bovine serum albumin (BSA). Invasion assays were then performed using BioCoat Matrigel Invasion Chambers (Corning Life Sciences, Tewksbury, MA) according to the manufacturer’s instructions. Briefly, cells (2.5 × 10 ^4^ cells) suspended in 0.5 ml of serum-free medium containing 0.1 % BSA were added to the upper chamber of Matrigel-coated filter inserts, and 0.75 ml of fresh medium containing 10 % FCS was added to the bottom well as a chemoattractant. The chambers were then incubated for 18 or 72 h. In assays using HeLaS3 cells, the cells in the upper chamber were treated with 200 nM PMA during the assay. After incubation, migrated cells on the underside of the filter were fixed and stained with 20 % (vol/vol) methanol containing 0.1 % (wt/vol) crystal violet powder for 5 min at room temperature. After extensive washing in PBS, the filters were photographed to count the number of migrated cells on the underside in 30 randomly selected fields.

### Gelatin zymography

At 32 h post-transfection, cells were serum starved for 16 h prior to being incubated in fresh serum-free medium containing the indicated reagents for further 7 or 24 h. To measure MMP activities in conditioned media, conditioned media were collected, cleared by centrifugation, normalized to cell number, mixed with non-reducing Laemmli’s sample buffer, and subjected to electrophoresis in a 10 % SDS-PAGE gel containing 0.1 % (wt/vol) gelatin. To measure MMP activities in cells, cells were washed with cold PBS twice and lysed in a lysis buffer [50 mM Tris/HCl pH 7.4, 150 mM NaCl, 1 × protease inhibitor cocktails (Roche Applied Science), 1 × phosphatase inhibitor mixture (Calbiochem), and 1 % NP-40] on ice for 10 min. Cell lysates were clarified by centrifugation at 10,000 × g for 10 min at 4 °C and the supernatant was collected. Protein lysate concentration was determined using the DC protein assay kit (Bio-Rad). The collected supernatant was mixed with non-reducing Laemmli’s sample buffer, followed by electrophoresis in a 10 % SDS-PAGE gel containing 0.1 % (wt/vol) gelatin. After electrophoresis, the gels were incubated in a renaturing buffer (2.5 % Triton X-100) with gentle agitation for 1 h at 22 °C, washed with distilled water three times, and incubated in a developing buffer (50 mM Tris/HCl pH 7.4, 150 mM NaCl, and 10 mM CaCl _2_) for 24 h at 37 °C to allow digestion of the gelatin. Finally, the gels were stained with Coomassie brilliant blue and destained in 45 % (vol/vol) methanol and 1 % (vol/vol) acetic acid to remove excess dye. The relative density of each gelatinolytic band was quantified using LAS-1000 plus gel documentation system (Fuji Film Co.).

### Reverse transcription (RT)-PCR

Total cellular RNA was isolated from cells using NucleoSpin RNA II (Takara, Shiga, Japan) according to the manufacturer’s instructions. Quantitative RT-PCR was performed using a light cycler nano (Roche Applied Science). Forward and reverse primers were as follows: human MMP-2, CCCCAAAACGGACAAAGAG and CTTCAGCACAAACAGGTTGC; human MMP-9, GAACCAATCTCACCGACAGG and GCCACCCGAGTGTAACCATA; human MMP-14, GCCTTGGACTGTCAGGAATG and AGGGGTCACTGGAATGCTC; human TIMP-1, CTGTTGTTGCTGTGGCTGAT and AACTTGGCCCTGATGACG; human TIMP-2, GAAGAGCCTGAACCACAGGT and CGGGGAGGAGATGTAGCAC; human GAPDH; CCTGTTCGACAGTCAGCCG and CGACCAAATCCGTTGACTCG; and human 18S rRNA; GCAATTATTCCCCATGAACG and GGGACTTAATCAACGCAAGC.

### Analysis of mRNA turnover

HT1080 cells were switched to fresh serum-free medium containing 1 μg/ml actinomycin D (Sigma-Aldrich) to inhibit transcription at 48 h post-transfection. HeLaS3 cells were pre-treated with 200 nM PMA for 24 h to promote accumulation of MMP-9 mRNA at 24 h post-transfection and subsequently switched to fresh serum-free medium containing 1 μg/ml actinomycin D to inhibit transcription. Samples were collected starting 30 min after actinomycin D treatment and at the indicated periods of time. Samples were assayed for MMP-9 mRNA and 18S rRNA mRNA levels by RT-PCR.

### MTT assay

Cells were seeded into a 96-well plate at a density of 2 × 10 ^3^ cells/well in triplicates. At 24, 48, and 72 h after transfection, 10 μl of MTT solution (Dojindo, Kumamoto, Japan) was added into each well. After incubation at 37 °C for 2 h, the OD at 450 nm was analyzed on a plate reader.

### Statistical analysis

Each experiment was independently repeated at least three times. The student’s *t*-test was used to determine the statistical significance of the value differences between experimental and control groups. Values of *p* less than 0.05 were considered significant.

## Results

### TSG101 depletion promotes cell invasion of HT1080 cells

**Fig. 1:**
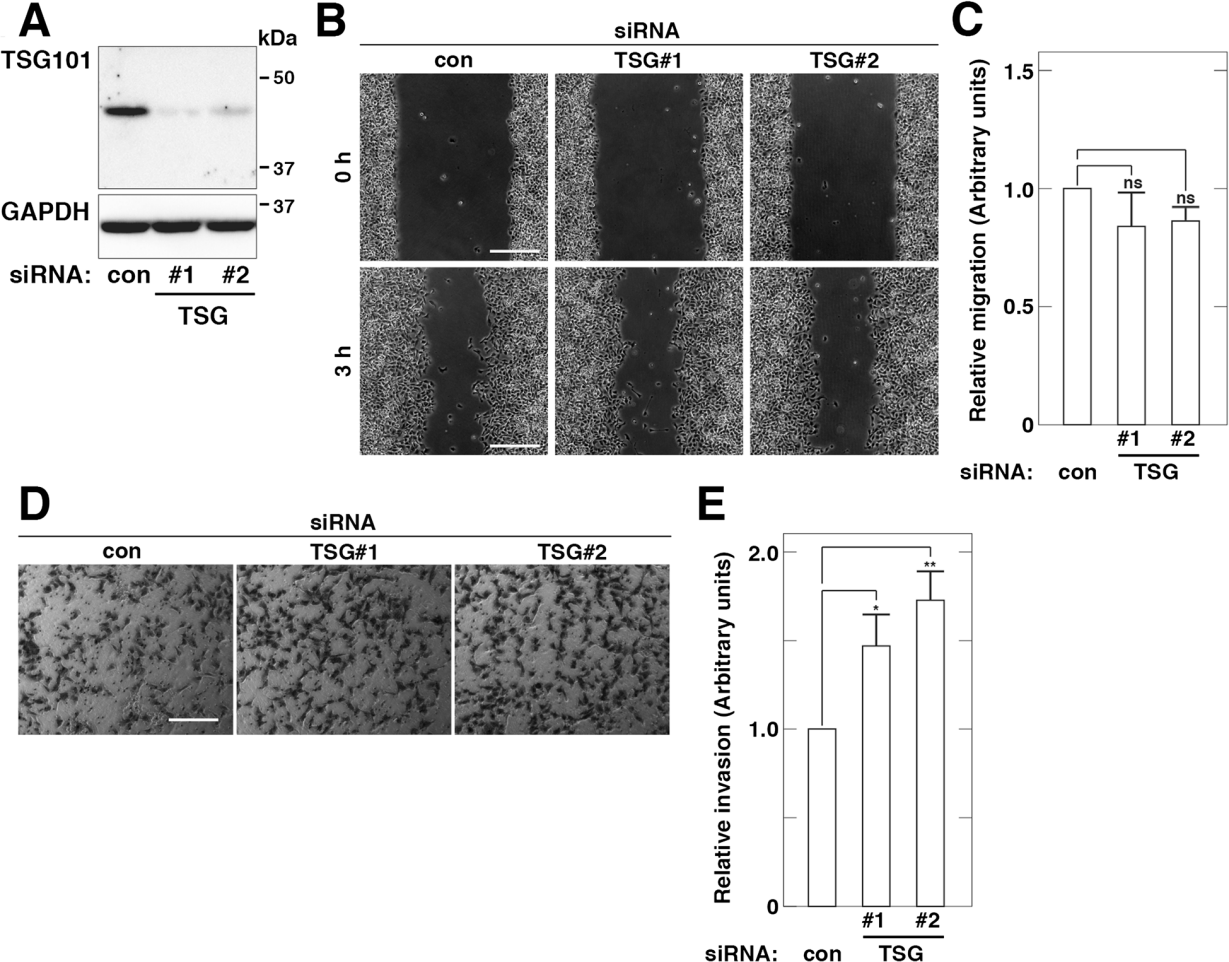
TSG101 depletion promotes cell invasion of HT1080 cells. **a**. Depletion of TSG101 by siRNA. Total cell lysates of cells transfected with control (con) or TSG101 (TSG#1 or #2) siRNA were analyzed by western blot using the indicated antibodies. **b**–**c**. Cell migration of TSG101-depleted cells. Confluent cells transfected with control (con) or TSG101 (TSG#1 or #2) siRNA were scratched and incubated in fresh serum-free medium for 3 h (**b**). Scale bars, 200 μm. Cell migration into the wound area in (**b**) was quantified (**c**). Relative migration activities of the cells transfected with TSG101 (TSG#1 or #2) siRNA are expressed as the proportion of migration of the cells transfected with control (con) siRNA. **d**–**e**. Cell invasion of TSG101-depleted cells. Cells transfected with control (con) or TSG101 (TSG#1 or #2) siRNA were allowed to invade for 18 h (**d**). Scale bars, 200 μm. Cell invasion through the filter in (**d**) was quantified (**e**). Relative invasion activities of the cells transfected with TSG101 (TSG#1 or #2) siRNA are expressed as the proportion of infiltration of the cells transfected with control (con) siRNA. The blots and images shown are representative of three independent experiments. The results shown are the means ± S.D. of three independent experiments. * *p* < 0.05; **, *p* < 0.005; ns, not significant, by a Student’s *t*-test

To explore the roles of TSG101 as a tumor susceptibility gene, we used RNAi to examine whether TSG101 is involved in tumor cell biological behaviors such as migration and invasion in HT1080 fibrosarcoma cells. Western blot analysis confirmed that targeted knockdown of TSG101 led to decreased levels of TSG101 expression (Fig. 1a). First, we examined the effect of TSG101 depletion on cell migration using a wound healing assay and found that depletion of TSG101 using TSG#1 or TSG#2 siRNA duplexes had no impact on cell migration (Fig. 1b, c). Next, we examined the effect of TSG101 depletion on cell invasion using a Transwell invasion assay. Depletion of TSG101 using TSG#1 or TSG#2 siRNA duplexes led to increased numbers of migrated cells on the underside of the filter (Fig. 1d, e), suggesting that TSG101 is involved in cell invasion of HT1080 cells.

### TSG101 depletion leads to increased levels of MMP-9 expression in HT1080 cells

**Fig. 2:**
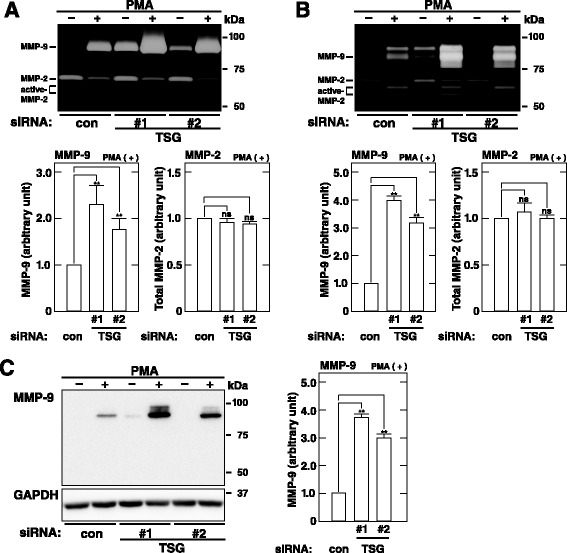
TSG101 depletion leads to increased secretion and expression of MMP-9 in HT1080 cells. **a**. MMP-9 secretion in TSG101-depleted cells. **b**–**c**. MMP-9 expression in TSG101-depleted cells. Cells transfected with control (con) or TSG101 (TSG#1 or #2) siRNA were incubated in fresh serum-free medium containing or not 200 nM PMA for 7 h. MMPs in conditioned media (**a**, top) and cell lysates (**b**, top) were measured using gelatin zymography. The amounts of total MMP-2 (MMP-2 + active-MMP-2) and MMP-9 in the conditioned media (**a**, bottom) and cell lysates (**b**, bottom) of PMA-treated cells were quantified and are expressed as arbitrary units. The amount of each MMP in the conditioned media and cell lysates of the cells transfected with control (con) siRNA is individually set to 1.0. Cell lysates were analyzed by western blot using the indicated antibodies (**c**, left). The amount of MMP-9 in PMA-treated cells was quantified and is expressed as arbitrary units (**c**, right). The amount of MMP-9 in the cells transfected with control (con) siRNA is set to 1.0. The blots and gels shown are representative of three independent experiments. The results shown are the means ± S.D. of three independent experiments. **, *p* < 0.005; ns, not significant, by a Student’s *t*-test

Gelatinases such as MMP-2 and MMP-9 play a crucial role in tumor cell aggressiveness such as invasion and metastasis [27–30]. We first used gelatin zymography to examine whether TSG101 is involved in secretion and expression of these MMPs in HT1080 cells. Depletion of TSG101 using TSG#1 or TSG#2 siRNA duplexes led to significantly increased levels of baseline MMP-9 secretion but did not impact baseline MMP-2 secretion (Fig. 2a). Stimulation of HT1080 cells by PMA induces enhanced MMP-9 secretion and MMP-2 activation [39, 41]. Depletion of TSG101 using TSG#1 or TSG#2 siRNA also led to significantly increased levels of PMA-induced MMP-9 secretion, but did not affect PMA-induced MMP-2 activation (Fig. 2a). Moreover, depletion of TSG101 using TSG#1 or TSG#2 siRNA duplexes led to significantly increased levels of MMP-9 expression but not MMP-2 expression in cells regardless of treatment with PMA (Fig. 2b). To explore whether TSG101 depletion leads to increased levels of MMP-9 protein in cells, we next performed western blotting experiments. Depletion of TSG101 using TSG#1 or TSG#2 siRNA duplexes led to significantly increased levels of MMP-9 protein at least in PMA-treated cells (Fig. 2c). Together, these results indicate that TSG101 depletion leads to increased MMP-9 protein levels and thereby enhances MMP-9 secretion in HT1080 cells.

### TSG101 depletion does not affect MMP-9 degradation in HT1080 cells

At least two possibilities could explain the increased levels of MMP-9 expression in TSG101-depleted cells: one is inhibition of MMP-9 degradation, and the other is enhancement of MMP-9 production. We first examined whether inhibition of proteasomal or lysosomal degradation leads to increased levels of secretion and expression of MMP-9 in HT1080 cells. Treatment with proteasome inhibitor MG132 or lysosome inhibitor bafilomycin A1 did not enhance MMP-9 secretion in control cells to the levels seen in TSG101-depleted cells regardless of treatment with PMA (Additional file 1: Figure S1A). Moreover, treatment with these inhibitors did not increase MMP-9 expression in control cells to the levels seen in TSG101-depleted cells regardless of treatment with PMA (Additional file 1: Figure S1B). The precise reason why bafilomycin A1 inhibited MMP-9 secretion is not known. However, since the NF-κB signaling pathway is strongly involved in activation of MMP-9 mRNA transcripts in HT1080 cells [43, 46] and since the proteasomal degradation of Iκ-B required for the activation of the NF-κB signaling pathway is inhibited by MG132 [47], it is likely that MG132 inhibits the NF-κB signaling pathway and thereby inhibits MMP-9 expression, especially PMA-induced MMP-9 expression, in both control and TSG101-depleted cells. These results support the possibility that TSG101 depletion enhances MMP-9 production.

### TSG101 depletion enhances MMP-9 mRNA expression in HT1080 cells

**Fig. 3:**
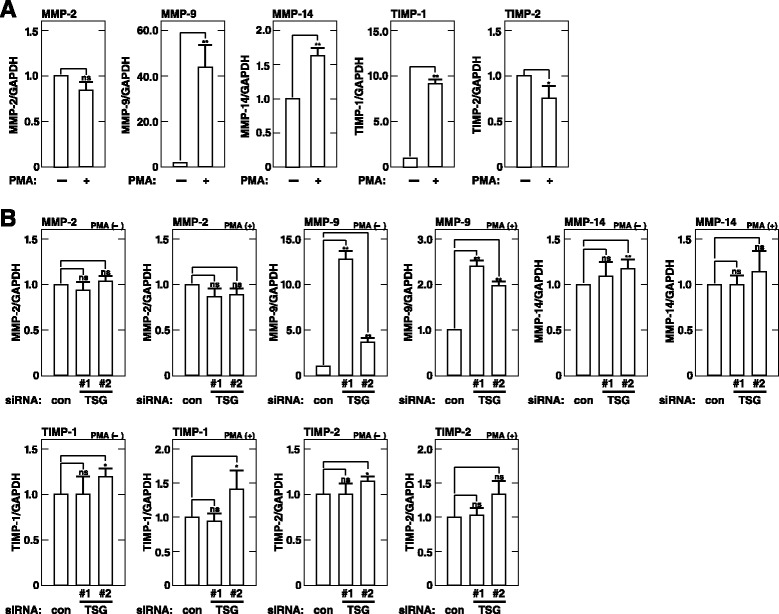
TSG101 depletion enhances MMP-9 mRNA expression in HT1080 cells. **a**. MMP-related gene mRNA expression. Subconfluent cells were serum starved for 16 h and subsequently incubated in fresh serum-free medium containing or not 200 nM PMA for 7 h. RT-PCR was performed to examine levels of the indicated mRNAs. The ratio of each MMP-related gene mRNA level relative to the GAPDH mRNA level is expressed as arbitraryunits. Each MMP-related gene level in non-treated cells is set to 1.0. **b**. MMP-related gene expression in TSG101-depleted cells. Cells transfected with control (con) or TSG101 (TSG#1 or #2) siRNA were incubated in fresh serum-free medium containing or not 200 nM PMA for 7 h. RT-PCR was performed to examine levels of the indicated mRNAs. The ratio of each MMP-related gene mRNA level relative to the GAPDH mRNA level is expressed as arbitrary units. Each MMP-related gene mRNA level in the cells transfected with control (con) siRNA is set to 1.0 individually in non- and PMA-treated cells. The results shown are the means ± S.D. of three independent experiments. * *p* < 0.05; **, *p* < 0.005; ns, not significant, by a Student’s *t*-test

MMP-9 production is regulated at the levels of gene transcription, mRNA stability, and translation [35–37]. We used RT-PCR to examine whether TSG101 depletion affects MMP-9 mRNA levels in HT1080 cells. Upon treatment with PMA, levels of MMP-9, MMP-9 inhibitor TIMP-1, and MMP-2 activator MMP-14 mRNAs were increased but those of MMP-2 and MMP-2 inhibitor TIMP-2 mRNAs were slightly decreased (Fig. 3a). Depletion of TSG101 using TSG#1or TSG#2 siRNA duplexes led to significantly increased levels of MMP-9 mRNA but had little impact on mRNA levels of any other examined molecules regardless of treatment with PMA (Fig. 3b). Depletion of TSG101 using TSG#1 siRNA duplexes led to more increased levels of MMP-9 mRNA than that using TSG#2 siRNA duplexes, consistent with the results in Fig. 2 showing that depletion of TSG101 using TSG101#1 siRNA duplexes led to more increased levels of secretion and expression of MMP-9 than that using TSG101#2 siRNA duplexes. These results demonstrate that TSG101 depletion specifically leads to significantly increased levels of MMP-9 mRNA in HT1080 cells.

### TSG101 depletion does not enhance the activities of the signaling pathways required for MMP-9 secretion in HT1080 cells

**Fig. 4:**
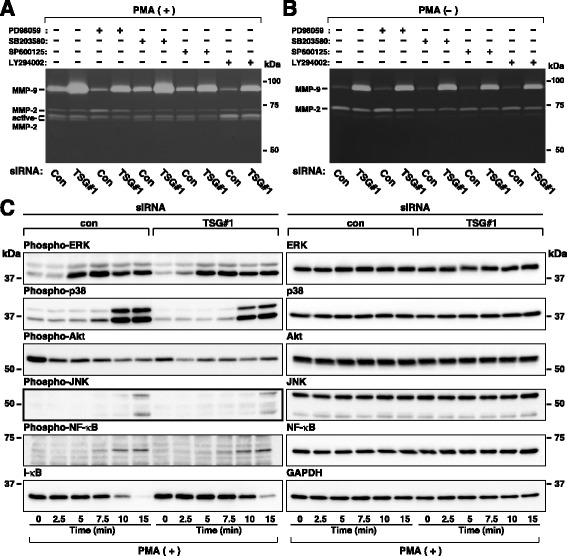
TSG101 depletion has little impact on the signaling pathways in PMA-stimulated HT1080 cells. Cells transfected with control (con) or TSG101 (TSG#1) siRNA were incubated in fresh serum-free medium containing the indicated reagents. **a**–**b**. Cells were incubated with 50 μM PD98059, 10 μM SB203580, 50 μM SP600125, or 50 μM LY294002 in fresh serum-free medium containing or not 200 nM PMA for 7 h. MMPs in conditioned media from PMA-treated (**a**) and non-treated (**b**) cells were measured using gelatin zymography. **c**. Cells were incubated in fresh serum-free medium containing 200 nM PMA for the indicated periods of time. Cell lysates were analyzed by western blot using the indicated antibodies. The blots and gels shown are representative of three independent experiments

Stimulation of tumor cells by PMA has been reported to lead to increased levels of MMP-9 secretion through activating several signaling pathways [39, 41, 42], raising the possibility that TSG101 depletion leads to increased levels of MMP-9 secretion through amplifying these signaling pathways. To evaluate this possibility, we first used gelatin zymography to examine the effect of TSG101 depletion on MMP-9 secretion in the presence or absence of chemical inhibitors of these signaling pathways in HT1080 cells. Treatment with the ERK inhibitor PD98095, p38 kinase inhibitor SB203580, JNK inhibitor SP600125, or PI3-K/Akt inhibitor LY294002 significantly reduced PMA-induced MMP-9 secretion in both control and TSG101-depleted cells, but did not decrease PMA-induced MMP-9 secretion in TSG101-depleted cells to the levels seen in control cells (Fig. 4a). Moreover, none of these inhibitors eliminated the increase in baseline MMP-9 secretion in TSG101-depleted cells (Fig. 4b). Taken together with the results shown in Additional file 1: Figure S1, these results demonstrate that the ERK, p38 kinase, JNK, PI3-K/Akt, and NF-κB signaling pathways are involved in PMA-induced MMP-9 secretion in HT1080 cells and suggest that TSG101 depletion does not enhance any of these signaling pathways required for PMA-induced MMP-9 secretion in HT1080 cells. To confirm this, we next performed western blotting experiments with phospho-specific antibodies. TSG101 depletion did not increase but instead slightly reduced activations of the ERK, p38 kinase, PI3-K/Akt, JNK, and NF-κB signaling pathways (Fig. 4c). Although the precise reason why TSG101 depletion reduced activation of these signaling pathways is not known, it is possible that these signaling pathways may be suppressed in a compensatory manner. Together, these results demonstrate that TSG101 depletion does not enhance MMP-9 secretion at least through amplifying any of these signaling pathways regardless of treatment with PMA.

### TSG101 does not affect MMP-9 mRNA stability in HT1080 cells

Several studies have reported that post-transcriptional mechanisms are involved in the regulation of MMP-9 mRNA levels [35, 36]. Therefore, to examine the possibility that TSG101 participates in post-transcriptional regulation of MMP-9 mRNA stability, we used an established approach to inhibit new gene transcription and measure MMP-9 mRNA decay over time [48]. There was not a significant difference in level of remaining MMP-9 or 18S rRNA mRNA at each time point between control and TSG101-depleted cells (Additional file 2: Figure S2A and B), suggesting that TSG101 does not participate in post-transcriptional regulation of MMP-9 mRNA stability in HT1080 cells.

### TSG101 modulates cell invasion of HT1080 cells in an MMP-9-dependent manner

**Fig. 5:**
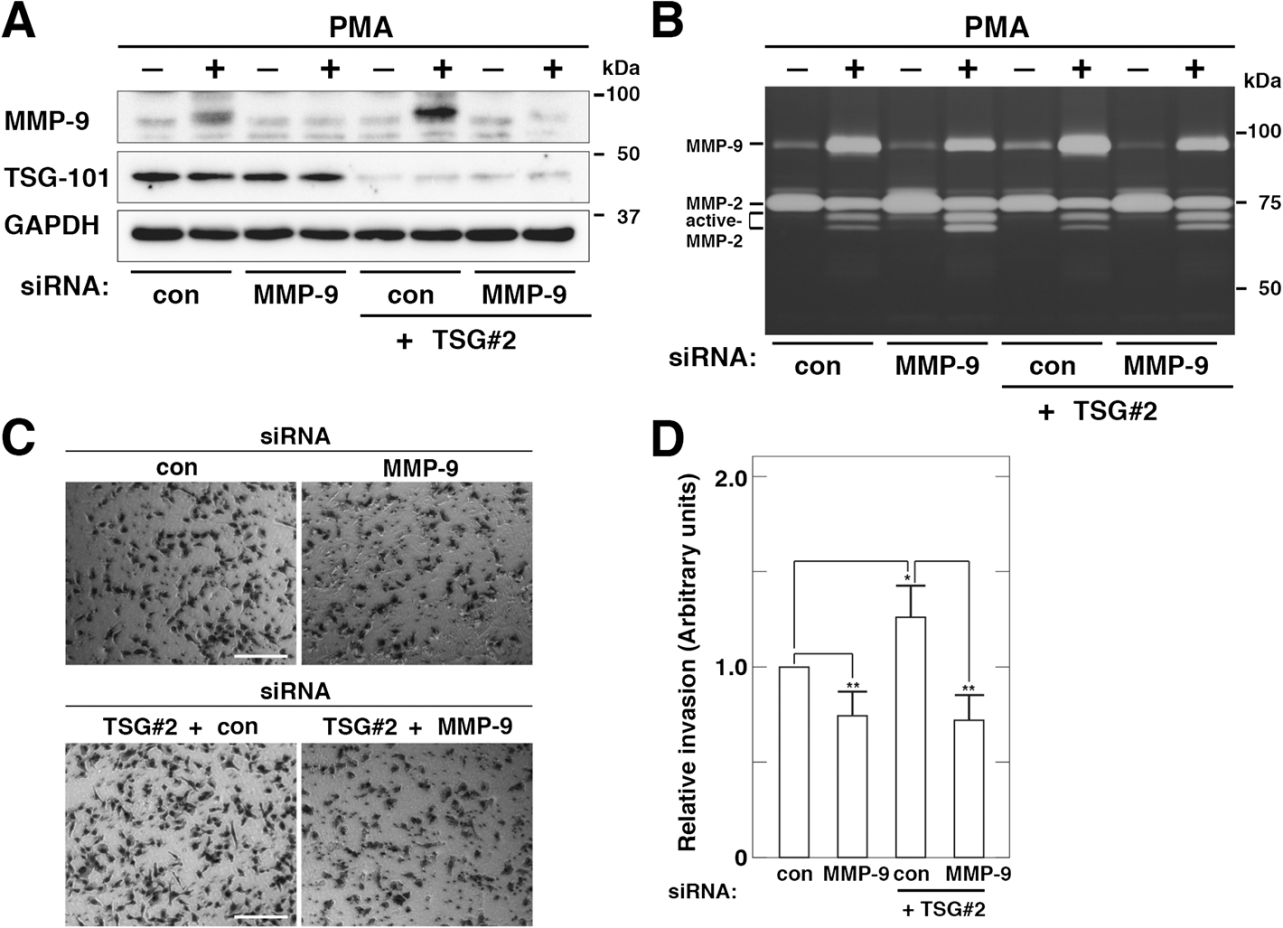
TSG101 modulates cell invasion of HT1080 cells in an MMP-9-dependent manner. Cells were transfected with control (con) or MMP-9 siRNA to deplete MMP-9, or with TSG101 (TSG#2) siRNA and control (con) or MMP-9 siRNA to simultaneously deplete TSG101 and MMP-9. **a**–**b**. Expression and secretion of MMP-9 in simultaneous TSG101- and MMP-9-depleted cells. Cells were incubated in fresh serum-free medium containing or not 200 nM PMA for 7 h. Cell lysates were analyzed by western blot using the indicated antibodies (**a**). MMPs in conditioned media were measured using gelatin zymography (**b**). **c**–**d**. Cell invasion of simultaneous TSG101- and MMP-9-depleted cells. Each of the transfected cells was allowed to invade for 18 h (**c**). Scale bars, 200 μm. Cell invasion through the filter in (**c**) was quantified (**d**). Relative invasion activities of the cells transfected with MMP-9 siRNA alone, and TSG101 (TSG#2) siRNA and control (con) or MMP-9 siRNA are expressed as the proportion of infiltration of the cells transfected with control (con) siRNA. The blots, gels, and images shown are representative of three independent experiments. The results shown are the means ± S.D. of three independent experiments. * *p* < 0.05; **, *p* < 0.005; ns, not significant, by a Student’s *t*-test

Our results strongly support the possibility that TSG101 is involved in cell invasion of HT1080 cells through regulating MMP-9 expression. To explore this possibility, we first examined whether MMP-9 is involved in cell invasion of HT1080 cells. Western blot analysis confirmed that targeted knockdown of MMP-9 by siRNA led to decreased levels of MMP-9 expression (Fig. 5a). MMP-9 depletion led to decreased levels of MMP-9 secretion regardless of treatment with PMA (Fig. 5b) and moreover led to decreased numbers of migrated cells on the underside of the filter (Fig. 5c. d), suggesting that MMP-9 is at least involved in cell invasion of HT1080 cells. MMP-9 depletion may partially inhibit cell invasion because MMP-2 secretion is more prominent in HT1080 cells than MMP-9 secretion. Next we examined the effect of simultaneous depletion of TSG101 and MMP-9 on cell invasion of HT1080 cells. Simultaneous depletion of TSG101 and MMP-9 reduced numbers of migrated cells on the underside of the filter to the levels seen in the cells treated with MMP-9 siRNA duplexes alone (Fig. 5c, d). Taken together with the results that depletion of neither TSG101 nor MMP-9 affected cell growth of HT1080 cells (data not shown), these results demonstrate that TSG101 is at least involved in cell invasion of HT1080 cells in an MMP-9-dependent manner.

### TSG101 depletion contrarily decreases MMP-9 mRNA expression in PMA-treated HeLaS3 cells

**Fig. 6:**
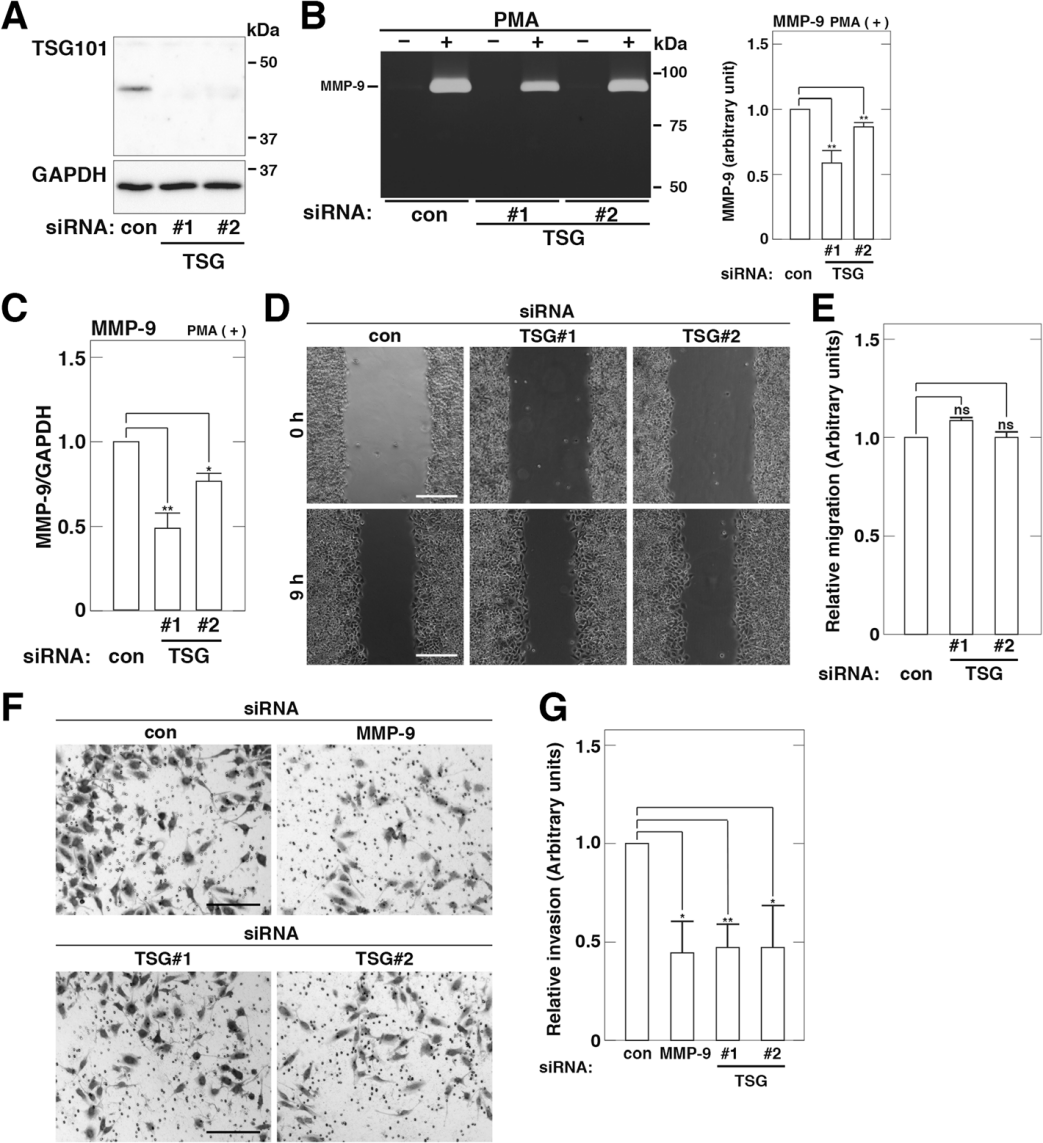
TSG101 depletion decreases MMP-9 secretion and reduces cell invasion in PMA-treated HeLaS3 cells. Cells were transfected with control (con), TSG101 (TSG#1 or #2), or MMP-9 siRNA. **a**. Depletion of TSG101 by siRNA. Total cell lysates were analyzed by western blot. **b**–**c**. MMP-9 secretion and MMP-9 mRNA expression in TSG101-depleted cells. Cells were incubated in fresh serum-free medium containing or not 200 nM PMA for 24 h. MMP9 in conditioned media was measured using gelatin zymography (**b**, left). The amount of MMP-9 in PMA-treated cells was quantified and is expressed as arbitrary units (**b**, right). The amount of MMP-9 in conditioned media of the cells transfected with control (con) siRNA is set to 1.0. Expression levels of MMP-9 and GAPDH mRNAs in PMA-treated cells were analyzed by RT-PCR (**c**). The ratio of MMP-9 mRNA level relative to the GAPDH mRNA level is expressed as arbitrary units. The MMP-9 mRNA level in the cells transfected with control (con) siRNA is set to 1.0. **d**–**e**. Cell migration of TSG101-depleted cells. Confluent transfected cells were scratched and incubated in fresh serum-free medium containing 200 nM PMA for 9 h (**d**). Scale bars, 200 μm. Cell migration into the wound area in (**d**) was quantified (**e**). Relative migration activities of the cells transfected with TSG101 siRNA are expressed as the proportion of migration of the cells transfected with control (con) siRNA. **f**–**g**. Cell invasion of TSG101-depleted cells. Each of the transfected cells was allowed to invade for 72 h (**f**). Scale bars, 200 μm. Cell invasion through the filter in (**f**) was quantified (**g**). Relative invasion activities of the cells transfected with TSG101 or MMP-9 siRNA are expressed as the proportion of infiltration of the cells transfected with control (con) siRNA. The blots, gels, and images shown are representative of three independent experiments. The results shown are the means ± S.D. of three independent experiments. * *p* < 0.05; **, *p* < 0.005; ns, not significant, by a Student’s *t*-test

To explore whether TSG101 depletion has the same effects in other cell lines as in HT1080 cells, we examined the effect of TSG101 depletion on MMP-9 mRNA expression in U2OS osteosarcoma and HeLaS3 cervical carcinoma cells. Western blot analysis confirmed that targeted knockdown of TSG101 in U2OS and HeLaS3 cells by siRNA led to decreased levels of TSG101 expression (Additional file 3: Figure S3A and Fig. 6a). TSG101 depletion exhibited the same effects on MMP-9 secretion, MMP-9 expression, and MMP-9 mRNA expression in U2OS cells (Additional file 3: Figure S3, b-e). However, depletion of TSG101 using TSG#1 siRNA duplexes contrarily led to decreased levels of MMP-9 secretion in PMA-treated HeLaS3 cells (Fig. 6b). Moreover, TSG101 depletion using TSG#1 siRNA duplexes proportionally led to decreased levels of MMP-9 mRNA in PMA-treated HeLaS3 cells (Fig. 6c). Since similar results were obtained using two sets of siRNA duplexes (TSG#1 and #2), it is not likely that the decrease in MMP-9 secretion and MMP-9 mRNA expression in PMA-treated cells is due to the off-target effect of siRNA treatment. Moreover, depletion of TSG101 using TSG#1 siRNA duplexes more effectively led to decreased levels of MMP-9 mRNA than that using TSG#2 siRNA duplexes, consistent with the results in Fig. 6b showing that depletion of TSG101 using TSG#1 siRNA duplexes more effectively led to decreased levels of MMP-9 secretion than that using TSG#2 siRNA duplexes. Together, these results raise the possibility that TSG101 might bidirectionally modulate cell invasion through regulating MMP-9 mRNA expression.

### TSG101 depletion reduces cell invasion of HeLaS3 cells

We next examined whether TSG101 depletion affects cell migration and cell invasion of HeLaS3 cells. Since the effect of TSG101 depletion on MMP-9 secretion in HeLaS3 cells was observed in the presence of PMA, the following experiments were performed in the presence of PMA. Depletion of TSG101 using TSG#1 or TSG#2 siRNA duplexes had no impact on cell migration of PMA-treated HeLaS3 cells (Fig. 6d, e). On the other hand, depletion of TSG101 using TSG#1 or TSG#2 siRNA duplexes led to decreased numbers of migrated PMA-treated cells on the underside of the filter (Fig. 6f, g). MMP-9 depletion using MMP-9 siRNA duplexes led to decreased numbers of migrated PMA-treated cells on the underside of the filter (Fig. 6f, g), suggesting that MMP-9 is also involved in cell invasion of HeLaS3 cells. Taken together with the results that depletion of neither TSG101 nor MMP-9 affected cell growth of HeLaS3 cells (data not shown), these results suggest that TSG101 may be implicated in the invasive potency of HeLaS3 cells as a tumor-enhancing gene.

### TSG101 depletion does not reduce the activities of the signaling pathways required for MMP-9 secretion in HeLaS3 cells

**Fig. 7:**
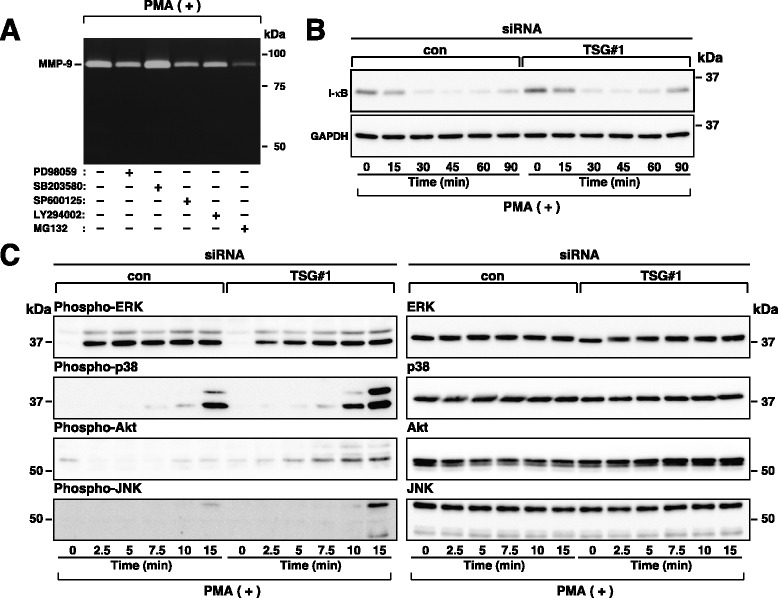
TSG101 depletion has little impact on the signaling pathways in PMA-stimulated HeLaS3 cells. Cells transfected with control (con) or TSG101 (TSG#1) siRNA were incubated in fresh serum-free medium containing the indicated reagents. **a**. Cells were incubated with 50 μM PD98059, 10 μM SB203580, 50 μM SP600125, 50 μM LY294002, or 10 μM MG132 in fresh serum-free medium containing 200 nM PMA for 24 h. MMP-9 in conditioned media was measured using gelatin zymography. **b**–**c**. Cells were incubated in fresh serum-free medium containing 200 nM PMA for the indicated periods of time. Cell lysates were analyzed by western blot using the indicated antibodies. The blots and gels shown are representative of three independent experiments

To explore the mechanism underlying decreased levels of PMA-induced MMP-9 mRNA in TSG101-depleted HeLaS3 cells, we first examined which of the signaling pathways are involved in PMA-induced MMP-9 secretion in HeLaS3 cells. Among various chemical inhibitors, PD98095, SP600125, LY294002, and MG132 significantly reduced PMA-induced MMP-9 secretion while SB203580 had no impact (Fig. 7a), suggesting that the ERK, JNK, PI3-K/Akt, and NF-κB signaling pathways may be at least involved in PMA-induced MMP-9 secretion in HeLaS3 cells. To explore whether TSG101 depletion inhibits PMA-induced activation of these signaling pathways in HeLaS3 cells, we next performed western blotting experiments with phospho-specific antibodies. Although phosphorylated NF-κB was not detected (data not shown), we speculate that TSG101 depletion did not affect the activation of the NF-κB pathway because TSG101 depletion did not affect I-κB degradation (Fig. 7b). Moreover, TSG101 depletion did not reduce but instead slightly enhanced activations of the p38 kinase, PI3-K/Akt, and JNK signaling pathways (Fig. 7c). Although the precise reason why TSG101 depletion promoted activation of these signaling pathways is not known, it is possible that these signaling pathways may be activated in a compensatory manner. These results suggest that TSG101 depletion does not decrease MMP-9 secretion at least through reducing any of these signaling pathways in PMA-treated HeLaS3 cells.

### TSG101 does not affect MMP-9 mRNA stability in HeLaS3 cells

To investigate whether TSG101 participates in post-transcriptional regulation of MMP-9 mRNA stability in HeLaS3 cells, we measured MMP-9 mRNA decay over time in HeLaS3 cells. However, because of the very low baseline levels of MMP-9 mRNA in HeLaS3 cells, it was necessary to first induce MMP-9 mRNA to detectable levels in these cells so that we could monitor the rate of mRNA turnover. There was not a significant difference in level of remaining MMP-9 or 18S rRNA mRNA at each time point between control and TSG101-depleted cells (Additional file 2: Figure S2C and D), suggesting that TSG101 does not participate in post-transcriptional regulation of MMP-9 mRNA stability in HeLaS3 cells.

## Discussion

In tumor invasion, degradation of ECM proteins by tumor cells disrupts this structure, enabling tumor cell invasion through the matrix [49]. The ability of tumor cells to invade into and migrate through their surrounding environment is directly correlated with the morbidity and mortality of all forms of cancer. Therefore, the mechanism of the degradation of ECM proteins by tumor cells has been intensively studied and evidence is emerging showing that MMPs play a central role in ECM degradation and cellular invasion [23–26]. Among the MMPs, MMP-9 is thought to play a pivotal role in the degradation of basement membrane collagen IV and therefore may mainly contribute to the invasive ability of various types of tumor cells [27–30]. Consistently, MMP-9 depletion reduced cell invasion of HT1080 and HeLaS3 cells, indicating a role for MMP-9 in cell invasion. On the basis of our present finding that TSG101 depletion promoted cell invasion and MMP-9 expression in HT1080 cells, but contrarily reduced these events in HeLaS3 cells, TSG101 may be implicated in the invasive potency of tumor cells through regulating the expression of MMP-9. As expected, the enhancement of cell invasion by TSG101 depletion was attenuated by simultaneous depletion of MMP-9 in HT1080 cells. Together, these data suggested that TSG101 participates in cell invasion in an MMP-9-dependent manner.

The expression and secretion of MMP-9 is regulated at several levels: transcription, mRNA stability, translation, protein degradation, and protein secretion [35–37]. We found that TSG101 depletion increased the levels of MMP-9 mRNA in HT1080 and U2OS cells, but contrarily decreased the levels of MMP-9 mRNA in HeLaS3 cells, and we also found that TSG101 depletion did not affect MMP-9 mRNA stability in either HT1080 or HeLaS3 cells. We did not explore the effect of TSG101 depletion on MMP-9 translation. However, since changes in MMP-9 protein levels were proportional to those in MMP-9 mRNA levels in TSG101-depleted cells, we speculate that TSG101 depletion does not affect this step. Together, our present results suggest that TSG101 might bidirectionally modulate the transcription of MMP-9 gene in different cell types.

The promoter of the human MMP-9 gene contains multiple functional cis-regulatory elements including AP-1, NF-κB, Sp-1, Ets-1, and Egr-1 elements, which are involved in baseline and induced transcriptional responses [43, 46, 50]. Several studies have shown that the transcription of MMP-9 is activated by several signaling pathways via these elements [40, 42, 50]. On the basis of our present finding that TSG101 depletion had little impact on any signaling pathways involved in PMA-induced MMP-9 secretion, it is not likely that TSG101 modulates the activities of the signaling pathways required for MMP-9 secretion. We have not yet elucidated the precise mechanism by which TSG101 participates in regulation of the transcription of MMP-9. However, TSG101 may directly or indirectly modulate the transcription of MMP-9 in the nucleus, based on the following observations. Previous reports showed that TSG101 acts as a transcriptional modulator to affect nuclear hormone receptor-mediated transcriptional activation [6, 9] and demonstrated that TSG101 interacts with and downregulates the promoter of p21, a tumor suppressor gene [7]. In synchronized cell lines, TSG101 is present in both the nucleus and Golgi complex during interphase, dispersed more generally throughout the cytoplasm in late S phase, and enriched in mitotic spindles and centrosomes during mitosis [15], implying that TSG101 plays diverse roles in both nucleus and cytoplasm.

The functions of TSG101 in carcinogenesis and tumor progression have been controversial. Besides cell invasion, we found that TSG101 depletion did not affect cell growth or cell migration of HT1080 or HeLaS3 cells, although it was reported that TSG101 depletion inhibits cell growth and cell migration of prostate cancer PC3, breast cancer MDA-MB-231, and breast cancer MCF-7 cells [19, 20]. We were unable to clarify the reason why TSG 101 depletion had no impact on cell growth or cell migration of HT1080 or HeLaS3 cells, but expect that the unprecedented link between TSG101 function and MMP-9 mRNA expression provides a clue to reveal the mechanism underlying the controversial functions of TSG101 in carcinogenesis and tumor progression. Further studies to unravel the precise mechanism by which TSG101 bidirectionally modulates the transcription of MMP-9 are necessary. Furthermore, we conceive that there may be a difference in pathological roles of TSG101 between tumor cell types derived from epithelial and mesenchymal tissues. TSG101 was originally identified as a tumor suppressor gene in mouse fibroblasts. HT1080 and U2OS cells, of which TSG101 depletion promoted cell invasion and increased MMP-9 expression, are derived from mesenchymal tissues. On the other hand, HeLaS3 cells, of which TSG101 depletion reduced cell invasion and decreased MMP-9 expression, are derived from epithelial tissues. PC3, MDA-MB-231, and MCF-7 cells, where TSG101 has been reported to function as a tumor-enhancing gene, are derived from epithelial tissues. Moreover, it has been recently reported that positive TSG101 expression is significantly associated with invasion of adenocarcinoma of the gallbladder [51]. Although further studies are necessary, TSG101 may play pathological roles as a tumor-enhancing gene in tumor cell types derived from epithelial tissues.

## Conclusion

In summary, we found that TSG101 bidirectionally modulated cell invasion through regulating MMP-9 mRNA expression in different cell types. TSG101, which was initially identified as a tumor suppressor gene, is also known to contrarily function as a tumor-enhancing gene [1]. To the best of our knowledge, our present findings are the first report providing a new insight into a putative mechanism by which TSG101 bidirectionally participates in carcinogenesis and tumor progression as a multifaceted gene in different cell types.

## Additional files

Additional file 1: Figure S1. TSG101 depletion does not affect MMP-9 degradation in HT1080 cells. Cells transfected with control (con) or TSG101 (TSG#1) siRNA were incubated with 10 μM MG132 or 100 nM bafilomycin A1 (BafA1) in fresh serum-free medium containing or not 200 nM PMA for 7 h. MMPs in conditioned media (**A**) and cell lysates (**B**) were measured using gelatin zymography. The gels shown are representative of three independent experiments. (PDF 1033 kb) Additional file 2: Figure S2. TSG101 depletion does not affect MMP-9 mRNA stability in either HT1080 or HeLaS3 cells. Stability of MMP-9 mRNA in TSG101-depleted HT1080 cells (**A** and **B**) and TSG101-depleted HeLaS3 cells (**C** and **D**). Cells transfected with control or TSG101 (TSG#1) siRNA were incubated in fresh serum-free medium containing 1 μg/ml actinomycin D for various time points and RT-PCR was performed to monitor turnover of MMP-9 mRNA. Expression levels of MMP-9 (**A** and **C**) and 18S rRNA (**B** and **D**) mRNAs were analyzed by RT-PCR. Results are plotted as the percentage of mRNA remaining relative to the starting amounts at 0 h individually in control and TSG101 (TSG#1) siRNA transfected cells. The results shown are the means ± S.D. of three independent experiments. Ns, not significant, by Student’s *t*-test. (PDF 351 kb) Additional file 3: Figure S3. TSG101 depletion leads to increased levels of MMP-9 mRNA in U2OS cells. (**A**) Depletion of TSG101 by siRNA. Total cell lysates of cells transfected with control (con) or TSG101 (TSG#1) siRNA were analyzed by western blot using the indicated antibodies. (**B**) and (**C**) Secretion and expression of MMP-9 in TSG101-depleted cells. Cells transfected with control (con) or TSG101 (TSG#1) siRNA were incubated in fresh serum-free medium containing or not 200 nM PMA for 7 h. MMPs in conditioned media (**B**) and cell lysates (**C**) were measured using gelatin zymography. (**D**) MMP-9 mRNA expression in PMA-treated cells. Subconfluent cells were serum starved for 16 h and subsequently incubated in fresh serum-free medium containing or not 200 nM PMA for 7 h. Expression levels of MMP-9 and GAPDH mRNAs were analyzed by RT-PCR. The ratio of MMP-9 mRNA level relative to the GAPDH mRNA level is expressed as arbitrary units. MMP-9 mRNA level in non-treated cells is set to 1.0. (**E**) MMP-9 mRNA expression in TSG101-depleted cells. Cells transfected with control (con) or TSG101 (TSG#1) siRNA were incubated in fresh serum-free medium containing or not 200 nM PMA for 7 h. The ratio of MMP-9 mRNA level relative to the GAPDH mRNA level is expressed as arbitrary units. MMP-9 mRNA level in the cells transfected with control (con) siRNA is set to 1.0 individually in non- and PMA-treated cells. The blots and gels shown are representative of three independent experiments. The results shown are means ± S.D. of three independent experiments. **, *p* < 0.005, by a Student’s *t*-test. (PDF 592 kb)

## Abbreviations

TSG101: Tumor susceptibility gene 101
MMPs: Matrix metalloproteinases
ECM: Extracellular matrix
TIMP: Tissue inhibitors of metalloproteinases
RNAi: RNA interference
FCS: Fetal calf serum
GAPDH: Glyceraldehyde-3-phosphate dehydrogenase
siRNA: Small interfering RNA
PBS: Phosphate buffered saline
PMA: Phorbol 12-myristate 13-acetate
BSA: Bovine serum albumin
RT: Reverse transcription
