# Supplementary figures and images for: TSG101, a tumor susceptibility gene, bidirectionally modulates cell invasion through regulating MMP-9 mRNA expression
Source: BMC Cancer. 2015 Nov 25;15:933. doi: 10.1186/s12885-015-1942-1 (PMC4660656; doi:10.1186/s12885-015-1942-1)

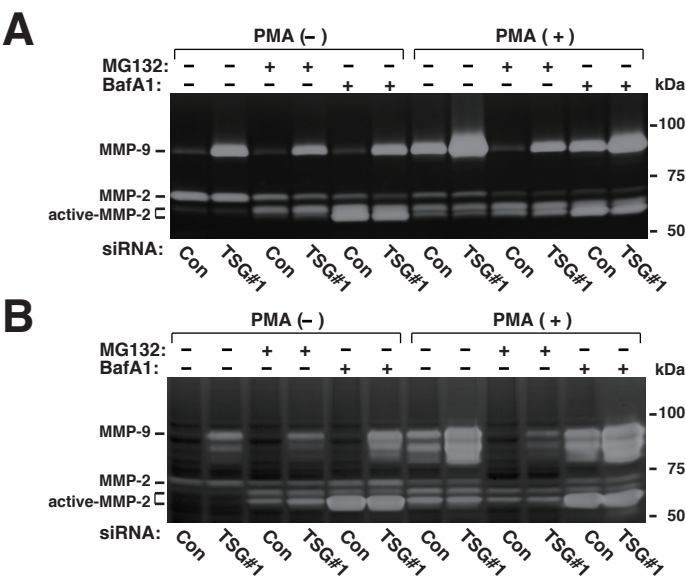

Supplement: Additional file 1: Figure S1. — TSG101 depletion does not affect MMP-9 degradation in HT1080 cells. Cells transfected with control (con) or TSG101 (TSG#1) siRNA were incubated with 10 μM MG132 or 100 nM bafilomycin A1 (BafA1) in fresh serum-free medium containing or not 200 nM PMA for 7 h. MMPs in conditioned media (A) and cell lysates (B) were measured using gelatin zymography. The gels shown are representative of three independent experiments. (PDF 1033 kb) [file 12885_2015_1942_MOESM1_ESM.pdf]

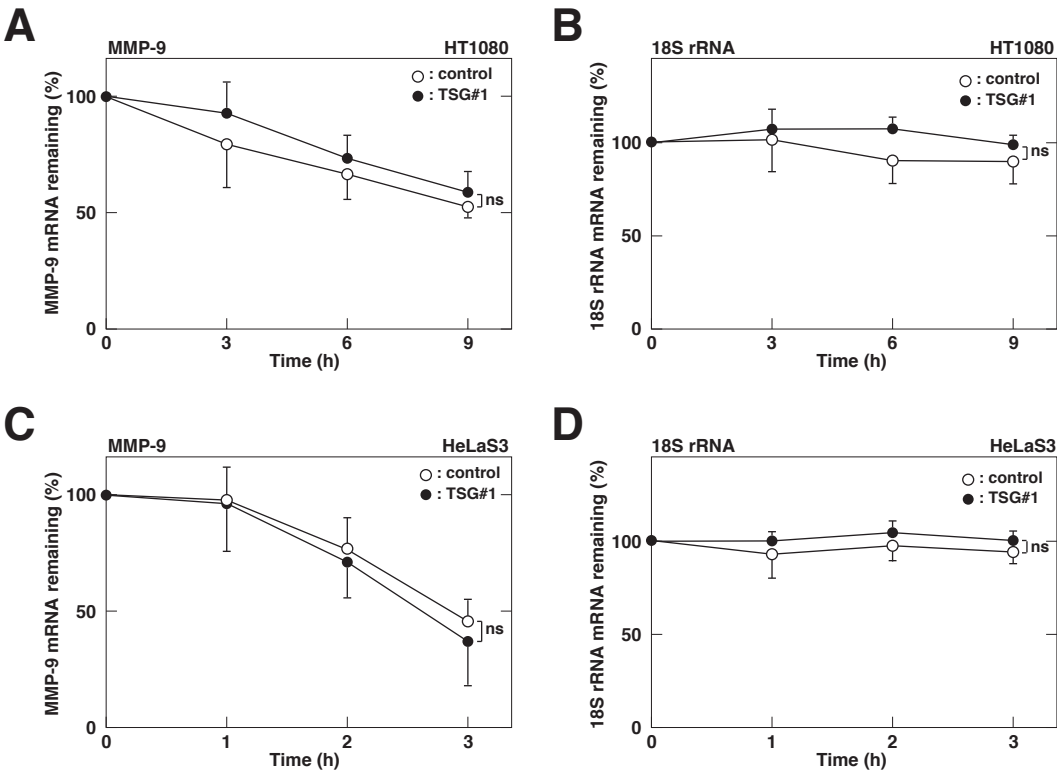

Supplement: Additional file 2: Figure S2. — TSG101 depletion does not affect MMP-9 mRNA stability in either HT1080 or HeLaS3 cells. Stability of MMP-9 mRNA in TSG101-depleted HT1080 cells (A and B) and TSG101-depleted HeLaS3 cells (C and D). Cells transfected with control or TSG101 (TSG#1) siRNA were incubated in fresh serum-free medium containing 1 μg/ml actinomycin D for various time points and RT-PCR was performed to monitor turnover of MMP-9 mRNA. Expression levels of MMP-9 (A and C) and 18S rRNA (B and D) mRNAs were analyzed by RT-PCR. Results are plotted as the percentage of mRNA remaining relative to the starting amounts at 0 h individually in control and TSG101 (TSG#1) siRNA transfected cells. The results shown are the means ± S.D. of three independent experiments. Ns, not significant, by Student’s t-test. (PDF 351 kb) [file 12885_2015_1942_MOESM2_ESM.pdf]

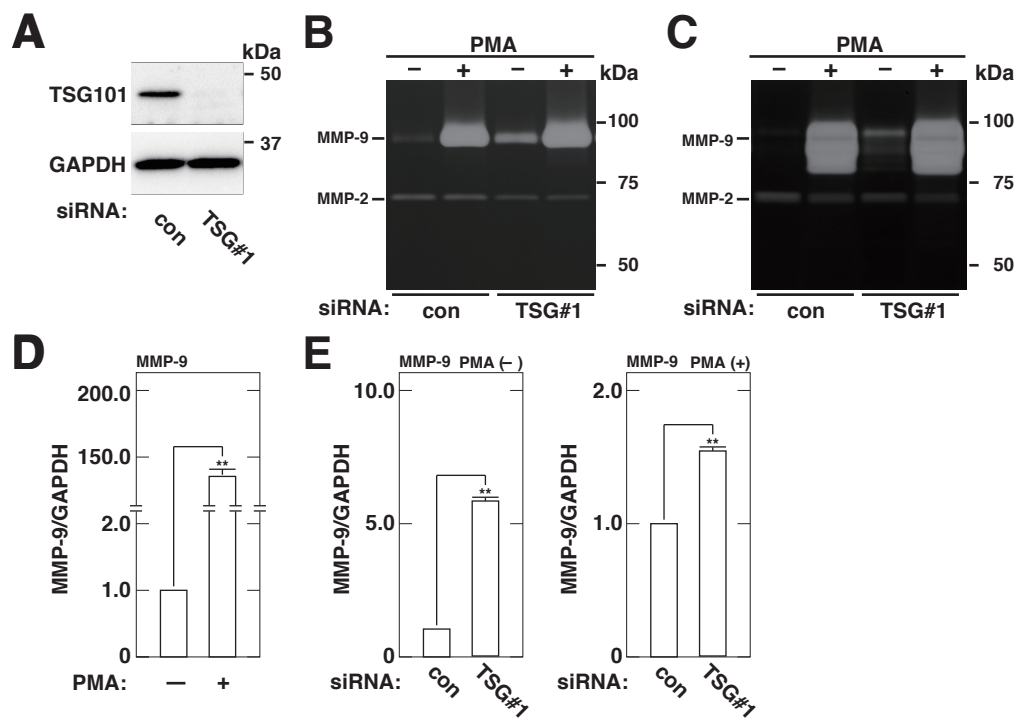

Supplement: Additional file 3: Figure S3. — TSG101 depletion leads to increased levels of MMP-9 mRNA in U2OS cells. (A) Depletion of TSG101 by siRNA. Total cell lysates of cells transfected with control (con) or TSG101 (TSG#1) siRNA were analyzed by western blot using the indicated antibodies. (B) and (C) Secretion and expression of MMP-9 in TSG101-depleted cells. Cells transfected with control (con) or TSG101 (TSG#1) siRNA were incubated in fresh serum-free medium containing or not 200 nM PMA for 7 h. MMPs in conditioned media (B) and cell lysates (C) were measured using gelatin zymography. (D) MMP-9 mRNA expression in PMA-treated cells. Subconfluent cells were serum starved for 16 h and subsequently incubated in fresh serum-free medium containing or not 200 nM PMA for 7 h. Expression levels of MMP-9 and GAPDH mRNAs were analyzed by RT-PCR. The ratio of MMP-9 mRNA level relative to the GAPDH mRNA level is expressed as arbitrary units. MMP-9 mRNA level in non-treated cells is set to 1.0. (E) MMP-9 mRNA expression in TSG101-depleted cells. Cells transfected with control (con) or TSG101 (TSG#1) siRNA were incubated in fresh serum-free medium containing or not 200 nM PMA for 7 h. The ratio of MMP-9 mRNA level relative to the GAPDH mRNA level is expressed as arbitrary units. MMP-9 mRNA level in the cells transfected with control (con) siRNA is set to 1.0 individually in non- and PMA-treated cells. The blots and gels shown are representative of three independent experiments. The results shown are means ± S.D. of three independent experiments. **, p < 0.005, by a Student’s t-test. (PDF 592 kb) [file 12885_2015_1942_MOESM3_ESM.pdf]
